# Fabrication and Characterization of Polyetherimide Electrospun Scaffolds Modified with Graphene Nano-Platelets and Hydroxyapatite Nano-Particles

**DOI:** 10.3390/ijms21020583

**Published:** 2020-01-16

**Authors:** Vassilis Kostopoulos, Athanasios Kotrotsos, Kalliopi Fouriki, Alexandros Kalarakis, Diana Portan

**Affiliations:** 1 Department of Mechanical Engineering and Aeronautics, University of Patras, Patras University Campus, GR-26504 Patras, Greece; akotrotso@mech.upatras.gr (A.K.); lfouriki@gmail.com (K.F.); diana.portan@mech.upatras.gr (D.P.); 2 Foundation of Research and Technology, Institute of Chemical Engineering Sciences (FORTH/ICE-HT), Stadiou Str., GR-26504 Patras, Greece; alexandros.kalarakis@uop.gr; 3 Department of Mechanical Engineering, School of Engineering, University of Peloponnese, M. Alexandrou 1, Koukouli, GR-26334 Patras, Greece

**Keywords:** solution electrospinning, scaffolds, bone tissue engineering, polyetherimide, GNPs, hydroxyapatite, SEM, TEM, contact angle, mechanical properties

## Abstract

Solution electrospinning process (SEP) is a versatile technique for generating non-woven fibrous materials intended to a wide range of applications. One of them is the production of fibrous and porous scaffolds aiming to mimic bone tissue, as artificial extracellular matrices (ECM). In the present work, pure and nano-modified electrospun polyetherimide (PEI) scaffolds have been successfully fabricated. The nano-modified ones include (a) graphene nano-platelets (GNPs), (b) hydroxyapatite (HAP), and (c) mixture of both. After fabrication, the morphological characteristics of these scaffolds were revealed by using scanning electron (SEM) and transmission electron (TEM) microscopies, while porosity and mean fiber diameter were also calculated. In parallel, contact angle experiments were conducted so that the hydrophilicity level of these materials to be determined. Finally, the mechanical performance of the fabricated scaffolds was investigated by conducting uniaxial tensile tests. Ιn future work, the fabricated scaffolds will be further utilized for investigation as potential candidate materials for cell culture with perspective in orthopedic applications.

## 1. Introduction

Solution electrospinning process is a simple, cost-effective, flexible, and versatile technique which produces non-woven fibrous materials from nano- to micro-scale. Generally, a SEP set-up consists of an injection pump, a syringe, a metallic needle, a high voltage power supply, and a grounded collector. During SEP, a polymer solution is pumped to the tip of the needle through the injection pump. By applying high voltage to the system, an electric field is generated between the tip of the needle and the collector plate. When the surface tension in the liquid droplet is overcome by the electric field, the droplet is distorted forming the Taylor cone [1]. The distortion leads to an electrically charged jet ejection that moves towards the collector, thus forming thin fibers. Using SEP, fibers with different morphological, physical, and mechanical properties can be fabricated by controlling the parameters which are applied to the system (i.e., voltage, solution feed rate, distance between the needle and collector, solution polymer concentration and electrical conductivity etc.) [2,3,4,5,6]. During the last decades, structures arose from SEP has attracted researchers’ interest in biomedicine since present ECM-like nanotopography, high porosity, and high specific area, thus helping the attachment, proliferation, and differentiation of cells. Besides that, SEP has proved useful in a wide range of applications such as air filtration [7,8], drug delivery [9,10], and oil/water separation [11]. In addition, SEP method is simple and more effective if compared to the others (i.e., rapid prototyping, melt extrusion, solvent evaporation, etc.), utilized for biomedical applications and especially for scaffold fabrication [12,13,14].

According to literature [15,16], SEP technique was proved to be promising in the field of bone tissue engineering since the fabricated structures simulate bone tissue and help in osteoblast culture. The skeletal system represents a body scaffold since it provides structure to the body, protects internal organs, and simultaneously acting as a calcium and phosphate reservoir [17]. It also, keeps remodeling and rebuilding throughout the lifetime and is responsible for support, locomotion, and load bearing [18]. However, the regeneration ability of a bone is strongly depending on the size of the defect that occurs. A wide range of materials have been utilized so far with an aim to replace the lost bone tissue (i.e., ceramics, polymers, metals, etc.) but none of them has been proven to be the ideal one. Most of the utilized materials, have the ability only to replace the structural function of the lost bone tissue and later must be removed as they cannot be resorbed. On the other hand, most of electrospun scaffolds are resorbable and promise to regenerate the lost tissue without the need for removal [17].

Based on previous published works [14,15], in order an artificial ECM substrate to be functional, it must have the appropriate porosity and fiber diameter to facilitate adhesion, proliferation, and differentiation of cells. Simultaneously, it must have adequate stiffness and strength to withstand forces during bone redevelopment. In addition, the applications of SEP implants made by degradable polymers are limited if compared to the metallic ones [19], due to the weak load-bearing capacity (i.e., to withstand large loads of bone). A variety of polymers (synthetic and natural) have been utilized so far in electrospun form for bone tissue engineering. Polycaprolactone (PCL) [20,21], is one of the most popular synthetic polymers utilized as implants of non-load-bearing parts (having low mechanical properties) for bone defect repair due to excellent biocompatibility and low toxicity. On the other hand, special engineering plastics—such as PEI—combine advantages of metals, good biocompatibility, and corrosion resistance. Also, PEI is characterized by remarkable mechanical properties as it presents similar elastic modulus to the trabecular bone and seems to be promising [22]. Several investigations exist in literature, in which PEI appears in electrospun form. In [23], Moon et al. fabricated well-aligned PEI electrospun fibers by optimizing the experimental parameters. In [24], Sung-Seen et al. achieve to enhance the interfiber bonding of PEI electrospun webs by applying heat treatment to the system, using a conventional oven. According to this investigation it was shown that after treatment the tensile properties were significantly improved.

PEI polymer has been studied so far, for its biocompatibility and has shown that allows the attachment and growth of cells [25,26]. For further improvement of mechanical and cellular performance of electrospun structures, GNPs and HAP inclusions have been incorporated into polymer fibers and further investigated [27,28]. Repanas et al. in [29], utilized multi-walled carbon nanotubes (MWCNTs) for fibers’ reinforcement. The incorporation of these nano-inclusions has shown to improve the electrospun structure’s characteristics. HA is a bio-ceramic known for its good biocompatibility, osteoconductivity, and mechanical properties [16,30,31,32]. HA constitutes the mineral component of bone that contributes to ~65% of the bone matrix by weight [32] and is an effective constituent for biomimetic materials that has been widely used in bone tissue engineering because of its good biocompatibility and osteoconductivity [32]. On the other hand, GNPs have attracted interest in biomedical applications because of their large surface area and mechanical strength. Graphene and its derivatives have been applied in many fields, including gene/drug delivery and tissue engineering, because of their unique physicochemical characteristics. These characteristics involve excellent electrical and thermal properties as well as high surface to volume ratio [32,33].

In the present work, pristine electrospun PEI scaffolds and nano-modified ones have been successfully fabricated. More precisely, pristine PEI and modified ones with (a) GNPs, (b) HAP, and (c) mixture of both (GNPs and HAP) have been fabricated for the needs of the present study. The aim of this work was to engineer and characterize PEI electrospun scaffolds (with and without nano-fillers) and study whether the nano-fillers improve the physical and mechanical properties of them. After fabrication, scaffold characteristics in terms of morphology have been examined by SEM and TEM microscopies. The obtained SEM images have been utilized to determine the mean fiber diameter, the porosity and the fiber distribution of each scaffold type. These characteristics significantly affect the mechanical performance and afterwards the cell activity during incubation and proliferation processes [14,15]. Furthermore, the hydrophilicity level of the fibrous scaffolds (both pristine and nano-filler containing) was determined by measuring the static water contact angle values. Finally, the apparent mechanical properties of the fabricated scaffolds have been assessed by conducting uniaxial tensile tests. In future work, the fabricated scaffolds (pristine and modified ones) will be further investigated as host matrix material for bone tissue regeneration. Finally, apart from bone tissue regeneration, PEI electrospun fibers could be utilized for other potential applications such as drug delivery [34], reinforcing of composite materials [35], filtration [36], and batteries [37].

## 2. Results and Discussion

### 2.1. Solution Electrospinning Process (SEP)

During SEP of all PEI solutions (pristine and nano-filler containing), an interesting phenomenon was observed as three-dimensional (3D) final fluffy structures were fabricated. This phenomenon is mainly attributed to the induction and polarization of the electrostatic charges, as opposing charge is gathered on the apex of the collected fibrous layer [18], which attracts the incoming electrospun fibers. The presence of opposing charge results in a preferential deposition of the new generated electrospun fibers on the existing fiber layer instead of the rest of the flat collector [18]. The main reason behind this phenomenon is dual; (a) due to the low surface resistivity of PEI fibers and (b) due to the high relative humidity of the surrounding environment (up to 80%) that led to further polarization of the THF/DMF solvent mixture (by humidity absorbance) [38]. Fibers with low surface resistivity quickly lose surface charges and allow incoming fibers to settle without much compaction [39]. On the other hand, at high relative humidity electrospun mats exhibit a fluffy, cotton-like texture indicating poor fiber bonding due to phase separation promoted by the presence of water. Thus, weak adhesion between the electrospun fibers has been achieved within the fabricated 3D structure [40].

3D electrospun structures have demonstrated more favorable for cell culture than the flat ones as provide larger spatial environment and have identical pore area. However, the fluffy nature of the structure induces poor mechanical strength because the fibers do not adhere to each other [23,24]. Based on that, the fabricated electrospun structures were further processed by hot-pressing upon appropriate conditions. Such conditions aimed to keep the fibrous structure of the scaffolds, identical pore area for cell culture and simultaneously to strengthen the fiber bonding for better stress transfer and improvement of the mechanical properties [24]. Hot-pressing process comprised a 5 min dwell at 60 °C under controlled loading of 1 MPa. After hot-pressing electrospun scaffolds were cooled down to RT, cut into small strips, and examined by SEM.

**Figure 1:**
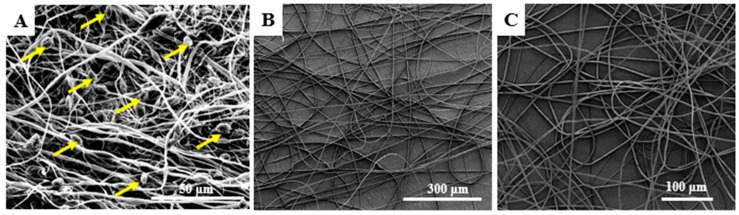
SEM images of electrospun scaffolds arose from a solution of THF/DMF (80/20 *w*/*w*) solvent mixture with pure PEI at the amount of (**A**) 10 wt %, (**B**) 15 wt %, and (**C**) 20 wt % respectively (yellow darts: bead formation).

In Figure 1, SEM images of electrospun scaffolds arose from three different PEI solutions with the following concentrations 10, 15, and 20 wt %, is shown after hot-pressing process. Comparing these images, it is shown that all samples keep its fibrous structure and will be further able to future support cell interaction. However, in Figure 1A (scaffold arose from solution with 10 wt % PEI) bead formation is noticed (yellow darts) along the electrospun fibers. Bead formation occurs due to (a) the low viscosity (inadequate polymer concentration), (b) the high surface tension of the polymer solution, and (c) the low charge density [41]. Generally, according to relative literature [42], beads’ presence breaks down the uniformity of scaffolds’ micro-structure and make difficult the attachment and proliferation of cells, as they create ‘internal defects’ which also results in reduction of their mechanical performance. Due to this fact, the polymer concentration into solution had to be increased for obtaining ultrafine fibers having better structure.

**Figure 2:**
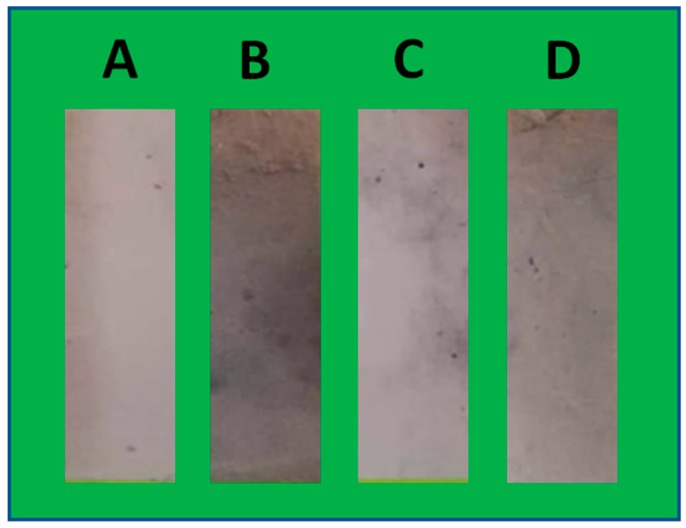
Illustration of the utilized (hot-pressed) scaffolds (**A**) pristine PEI, (**B**) PEI scaffold modified with GNPs, (**C**) PEI scaffold modified with HAP, and (**D**) PEI scaffold modified with GNPs and HAP.

Based on that, higher concentration PEI solutions (15 and 20 wt %) prepared in order SEP to be conducted. According to SEM images of Figure 1B,C, after electrospinning the obtained scaffolds seem to be uniform, homogeneous and cylindrical bead-less, consisting of dense network with no particular alignment. A well interconnected pore network structure is also shown. Finally, scaffolds arose from solution containing 15 wt %. PEI was selected for further investigation. Toward this direction, solutions containing (a) pure PEI, (b) pure PEI with 0.5 wt % GNPs, (c) pure PEI with 1 wt % HAP, and (d) pure PEI with 0.5 wt % GNP and 1 wt % HAP was prepared for electrospinning. Representative samples for each scaffold type after SEP are clearly illustrated in Figure 2.

### 2.2. Structural and Morphological Analysis

**Figure 3:**
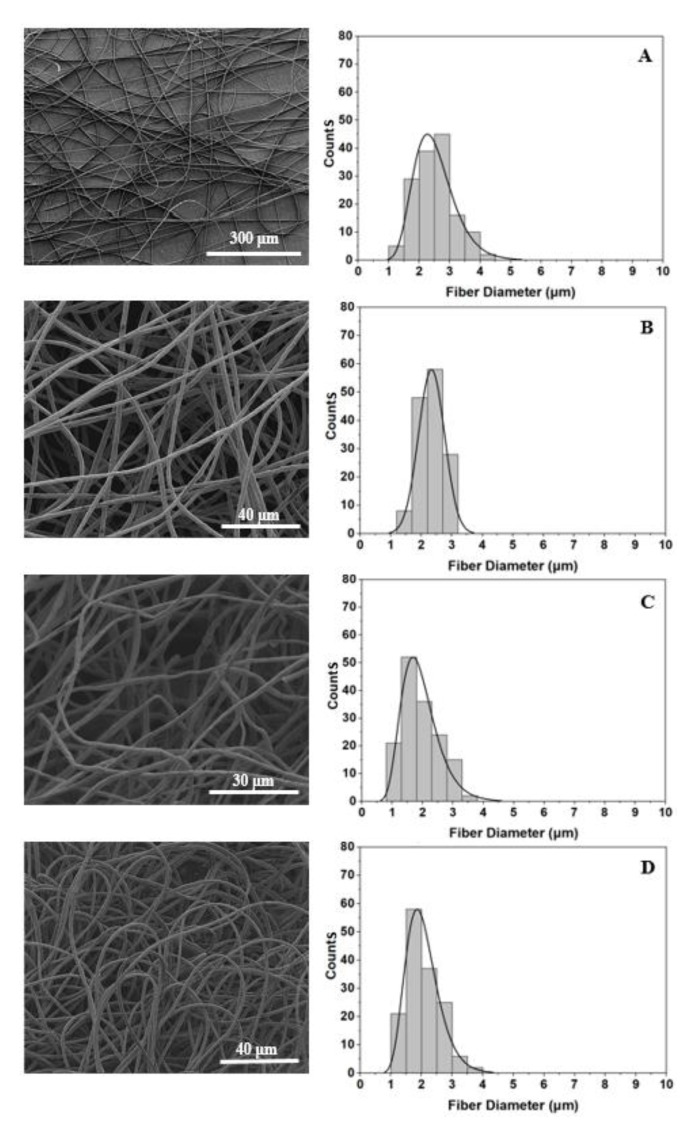
SEM images of (**A**) pristine PEI scaffold, (**B**) PEI scaffold modified with 0.5 wt % GNPs, (**C**) PEI scaffold modified with 1 wt % HAP, and (**D**) PEI scaffold modified with 0.5 wt % GNPs and 1 wt % HAP, together with histograms providing the fiber diameter distribution.

**Figure 4:**
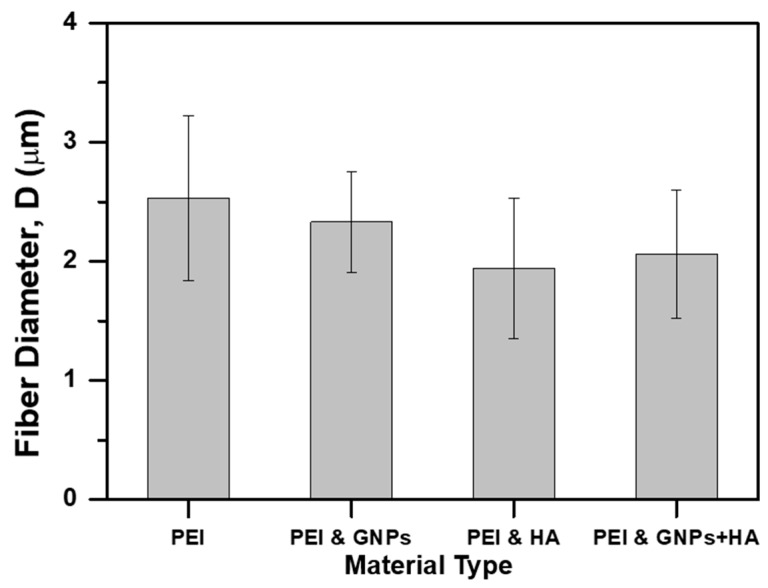
Bar chart diagram providing the mean fiber diameter and standard deviation (SSD) of the fabricated PEI scaffolds (pristine and nano-filler containing ones).

After SEP of all solutions, SEM examination was conducted to investigate the morphology of the fabricated scaffolds. Figure 3A–D correspond to pristine PEI, 0.5 wt % GNP modified, 1 wt % HAP modified, and 0.5 wt % GNP with 1 wt % HAP modified scaffolds respectively. According to SEM images of Figure 3, uniform and bead-less micro-fibers with 3D porous structures and random orientation have also been obtained after solution nano-modification (Figure 3B–D). Figure 3 also provides histograms containing the fiber diameter distribution for each scaffold type. On the other hand, bar chart of Figure 4 summarizes the value of mean fiber diameter for all scaffold types together with SSDs. Taking into consideration histograms of Figure 3 and bar charts of Figure 4, it was shown that the mean fiber diameter generally decreases by nano-modification, with scaffolds containing HAP to exhibit the lowest values. An analogous behavior was also observed in [43], in which supramolecular (SP) polymer scaffolds were fabricated and further investigated. More precisely, mean fiber diameter decreased from 2.53 ± 0.69 μm (pristine PEI), to 2.33 ± 0.42 μm for samples containing 0.5 wt % GNPs (7.9% reduction), to 1.94 ± 0.59 μm for samples containing 1 wt % HAP (23.3% reduction) and to 2.06 ± 0.54 μm for samples containing 0.5 wt % GNPs and 1 wt % HAP (18.6% reduction). Values obtained for all scaffold types exhibited lognormal distribution apart from GNP modified scaffold which exhibited normal one. The fiber diameter decrease by nanomodification was expected as solutions’ parameters (i.e., electrical conductivity) altered prior electrospinning [44]. Thus, nanofillers’ inclusion contributed to electrostatic charge build up during SEP. Finally, the quite large SSD values are common in electrospinning technique [28,43] as small changes of the electric field occur during the phenomenon.

**Figure 5:**
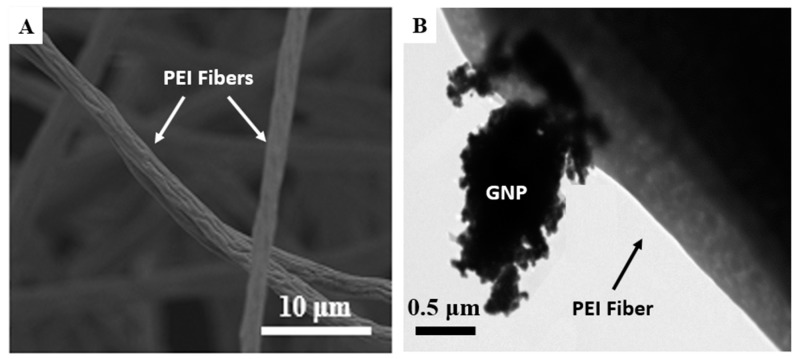
(**A**) Representative SEM image indicating the surface roughness of PEI electrospun fibers’ surface; (**B**) TEM image arose from PEI scaffold modified with 0.5 wt % GNPs, indicating the poor dispersibility of the nano-filler.

Apart from SEM examination of the fabricated PEI scaffolds, TEM examination was also conducted (see Figure 5B). According to representative SEM and TEM images of Figure 5A,B respectively, PEI electrospun fibers are non-transparent and presented surfaces with enhanced roughness. Based on that, reinforcements (i.e., nano-fillers) are not able to be visible. On the other hand, in TEM image of Figure 5B a GNP was caught to be outside the electrospun fiber. This behavior is attributed to the high electrostatic charges during SEP and to the high electrical conductivity of the nanofiller that led to poor dispersibility and further reinforcement of the entire fibrous structure. It is expected that such behavior will significantly affect the mechanical performance of the fabricated GNP-modified scaffold.

### 2.3. Porosity

**Figure 6:**
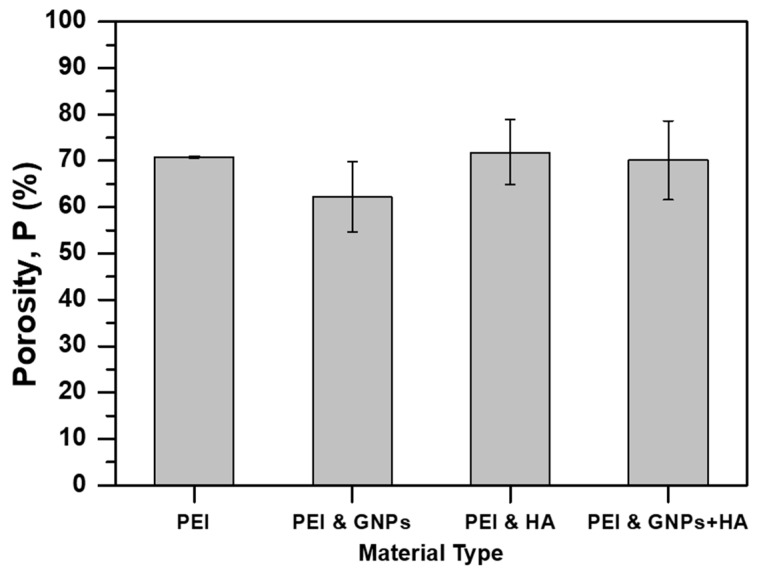
Bar chart diagram indicating the effect of nano-particle inclusion to the porosity (P) value of the fabricated scaffolds.

Nanomodification of scaffolds is also expected to have an impact on its final porosity value. Bar chart of Figure 6 provides the porosity value with respect to material type. According to this Figure, it is shown that all scaffold types presented similar porosity values (from 70.11% ± 0.08% to 71.81% ± 0.07%) apart from samples containing GNPs, which presented slightly reduced porosity by almost 12% (62.21% ± 0.07%) when compared to the porosity value of the pristine one (pure PEI scaffolds). Similar porosities for the most of scaffolds is mainly attributed to the common processing parameters (i.e., SEP critical parameters, heating and pressuring) that were applied to samples during fabrication. The slight reduction of the porosity for the GNP modified scaffolds is out of significance and was also observed in similar investigations of [43]. According to relative literature [45], the obtained porosity values are acceptable for the interconnectivity, the interaction and the proliferation process of cells.

### 2.4. Static Water Contact Angle Assay

**Figure 7:**
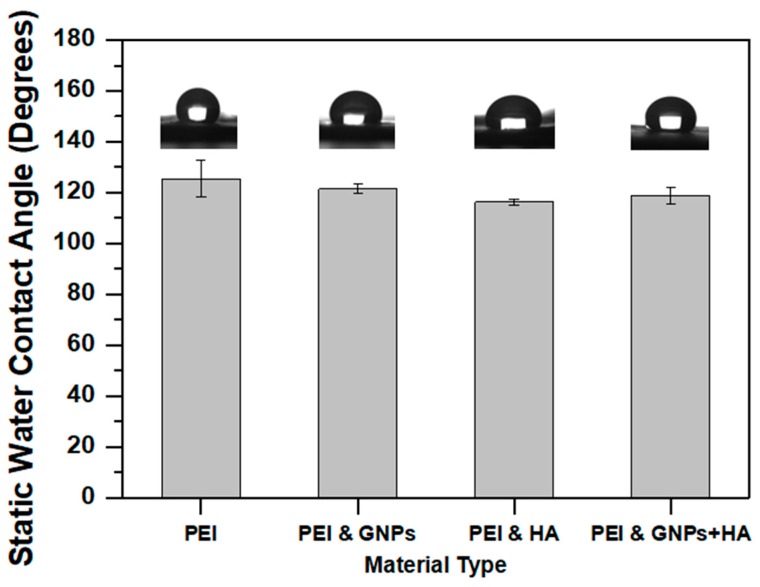
Bar chart diagram, providing the effect of nano-particle inclusion on the average contact angle value of the fabricated PEI scaffolds. Standard deviations (SSD) are provided.

In this section, a static contact angle assay is conducted with the aim to investigate the effect of nano-inclusions on the hydrophilicity level of PEI scaffolds. Bar chart of Figure 7, provides and compares the average contact angle values for all scaffold types. According to this Figure, all material sets presented static contact angle values above 90°, which indicates their hydrophobic nature. This behavior is mainly attributed to the presence of free polar molecules encounter in PEI chemical structure.

According to experimental results, it was shown that samples containing GNPs exhibited slightly reduced contact angle value by 3.2% (from 125.47 ± 7.15° to 121.37 ± 1.84°). In the presence of HAP nano-fillers the contact angle was reduced by 7.3% (from 125.47 ± 7.15° to 116.23 ± 1.06°) due to HAP hydrophilic nature. For samples containing both types of nanofillers contact angle value showed a reduction of 5.2% (from 125.47 ± 7.15° to 118.90 ± 1.06°). The same behavior was also observed in [43]. The small differences in contact angle values for all nano-modified scaffolds when compared to the pristine one (pure PEI), is attributed to the relative low concentration of nano-fillers (bellow 1.5 wt %) and further to the hot-pressing, which in its turn led to negligible differences in roughness among the examined materials. Small SSD values also confirm uniformity of the fabricated scaffolds.

### 2.5. Mechanical Properties

The apparent mechanical properties of the fabricated PEI scaffolds (nano-modified of not), were assessed by conducting uniaxial tensile tests. Nano-particles’ incorporation into fiber’s architecture by SEP is expected to have also an impact on scaffolds’ mechanical performance. Based on that, uniaxial tensile tests were carried out according to specifications described in Section 3.6.

**Figure 8:**
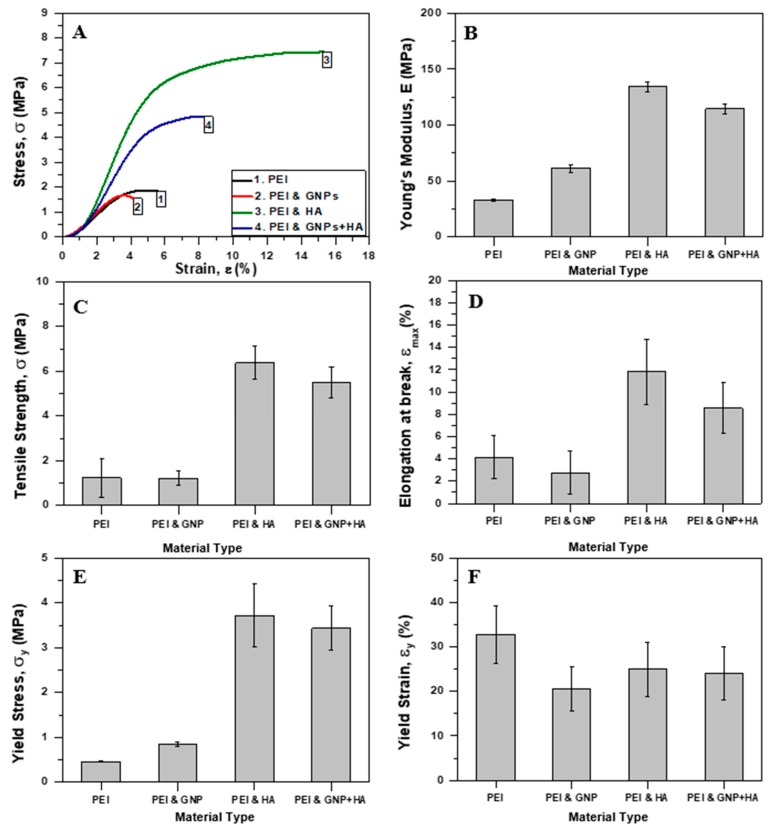
(**A**) Representative stress (σ) vs. strain (ε) % curves for pristine and nano-filler containing PEI scaffolds (with GNPs, HAP and mixture of both). Bar chart diagrams providing the mechanical properties of PEI scaffolds (pristine and nano-filler containing) that arose from uniaxial tensile tests; (**B**) Young’s modulus (E), (**C**) tensile strength (σ_max_), (**D**) elongation at break (ε_max_) %, (**E**) yield stress (σ_y_), and (**F**) yield strain (ε_y_) %. For all tensile properties standard deviations (SSD) are provided.

**Figure 9:**
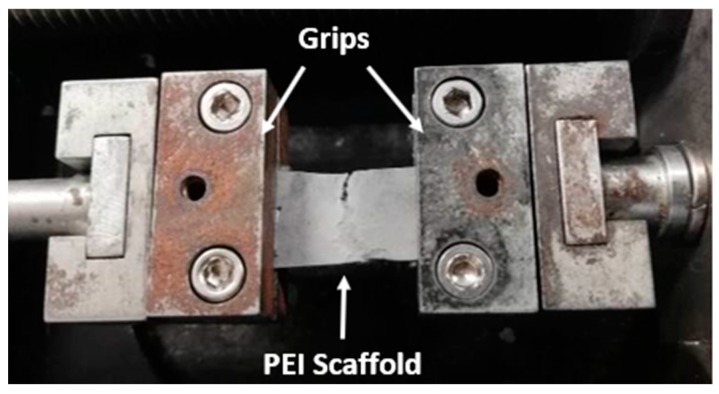
Snapshot during uniaxial tensile tests, indicating the brittle fracture of PEI membranes.

Figure 8A, illustrates representative apparent stress (σ)-strain (ε) % curves for pristine (pure PEI) and nano-modified scaffolds (with GNPs, HAP and mixture of both). For each scaffold type the apparent young’s modulus (E), tensile strength (σ_max_), strain at break, % (ε_max_, %), yield stress (σ_y_), and yield strain, % (ε_y_, %) values were calculated. All scaffold types exhibited typical yield behavior. An analogous behavior was also observed in [28] and in [43], in which Poly(methyl methacrylate) (PMMA) and SP scaffolds were fabricated and tested under the same conditions. Bar charts of Figure 8A–E provide average values of the apparent E, σ_max_, ε_max_ (%), σ_y_, and ε_y_ (%) respectively. All material sets presented brittle fracture as Figure 9 indicates.

According to experimental results shown in Figure 8, it is observed that the incorporation of GNPs did not affect significantly the σ_max_ value of PEI scaffolds, which presented a slight reduction by 2.4% (from 1.23 ± 0.86 MPa to 1.20 ± 0.31 MPa) and is out of significance. On the contrary E value significantly increased by 84% (from 33.27 ± 0.89 MPa to 61.34 ± 3.41 MPa). Furthermore, the ε_max_ % value decreased by 33% (from 4.15 ± 1.95% to 2.77 ± 0%), σ_y_ value increased by 85% (from 0.46 ± 0.01 MPa to 0.85 ± 0.04 MPa), while ε_y_ value remained constant (close to 1.39%). This behavior reveals the poor reinforcing effect of GNPs with host polymer material (PEI), that is attributed to (a) the absence of free hydroxyl groups in PEI chemical structure that led to poor adhesion between PEI and GNPs (hydroxyl groups would help the creation of chemical bonds between PEI and GNPs [22]), and (b) due to the SEP of the scaffolds [46]. Possibly, the quick solvent evaporation during SEP enhanced the phenomenon. This behavior is also confirmed by a TEM image, in which GNPs were detected outside the electrospun nano-fibers (see Figure 5B).

On the other hand, samples containing HAP showed entirely different behavior as exhibited significantly increased mechanical properties and the best mechanical performance. This behavior suggests that good adhesion between PEI polymer and HAP has been achieved together with good dispersion and absence of aggregations within the fibrous structure. The results also confirmed that the selected nanoparticles’ and concentration are effective and identical. More precisely, σ_max_ value increased by 417% (from 1.23 ± 0.86 MPa to 6.37 ± 0.75 MPa), E value by 304% (from 33.27 ± 0.89 MPa to 134.29 ± 4.48 MPa) and ε_max_% value by 184% (from 4.15 ± 1.95% to 11.80 ± 2.94%). σ_y_ and ε_y_% values were increased by 706% (from 0.46 ± 0.01 MPa to 3.71 ± 0.70 MPa) and 100% (from 1.39 ± 0.01% to 2.77 ± 0%) respectively. An analogous behavior was also observed in similar study of [47].

Based on the obtained results described above for scaffolds containing either GNPs or HAP, it was shown that scaffolds containing HAP exhibited the best mechanical performance although GNP itself is stiffer. Thus, it is understood that the adhesion between polymer and reinforcement within a hybrid system has critical effect on materials’ final mechanical properties. Perhaps, the utilization of other type of GNPs having lower lateral dimensions and/or the application of higher GNP concentration into the final solution lead to better results. Further research considering these aspects is proposed to be performed in future work, towards increasing the mechanical properties of the final scaffold by optimization. Potential problems during this research that are related to cell culture process (i.e., toxicity, etc.) should take also into consideration.

Finally, as it was expected, samples containing the mixture of both nano-inclusions exhibited intermediate tensile properties compared to the other two nano-modified scaffolds (containing GNPs or HAP). More precisely, σ_max_ value increased by 347% (from 1.23 ± 0.86 MPa to 5.50 ± 0.70 MPa), E value by 244% (from 33.27 ± 0.89 MPa to 114.46 ± 4.70 MPa) and ε (%) value by 106% (from 4.15 ± 1.95% to 8.55 ± 2.27%). σ_y_ and ε_y_ (%) values were increased by 645% (from 0.46 ± 0.01 MPa to 3.43 ± 0.49 MPa) and 115% (from 1.39 ± 0.01% to 2.99 ± 0.31%) respectively.

## 3. Materials and Methods

### 3.1. Raw Materials

The utilized PEI (trade name: ULTEM™ HU1000) was developed by SABIC (Riyadh, Saudi Arabia) and supplied by Westlake Plastics (Tourcoing, France). ULTEM™ HU1000 holds biocompatibility certifications (ISO 10993 and USP Class VI). PEI is an amorphous thermoplastic polymer, which exhibits excellent resistance to a wide range of chemicals and disinfectants and has a glass transition temperature (Tg) of about 217 °C. PEI based materials maintains its size and shape over a broad temperature range as tolerates a high amount of stress over extended periods of time. Tetrahydrofuran (THF, inhibitor-free for HPLC ≥ 99%) and N, N Dimethylformamide (DMF, anhydrous ≥ 99.8%), which played the solvent role, were utilized and purchased by Sigma Aldrich, Saint Louis, USA. Regarding the nano-fillers, four-layer GNPs and a synthetic ‘needle-like’ HAP were utilized. The obtained GNPs have lateral dimensions of 1–2 μm, average thickness ≤ 4 nm, surface area ≥ 750 m^2^/gr and purity ≥ 99% and were supplied by Cheap Tubes Inc., Cambridgeport, USA. The synthetic ‘needle-like’ HAP have an average length and thickness of 150 nm and 20 nm respectively, purity ≥ 97.5% and were purchased by Shanghai Xinglu Chemical Tech., Shanghai, China. In addition, the utilized HAP material contains many minerals (Mg ≤1.8%, Na ≤0.2%, Fe ≤0.08% and Al ≤0.1%) and was also utilized in a recent work by the authors [43].

### 3.2. Solution Preparation and Solution Electrospinning Process (SEP)

PEI was dissolved in a mixture of THF/DMF solvents (80/20 *w*/*w*), which was the most successful solvent combination for fiber formation according to previous study [48]. Such a balance between the two solvents is necessary to obtain dry final coating structures. Then, solutions with three different polymer concentrations (10, 15, and 20 wt %) were prepared and examined in order the optimal one to be identified. Afterwards, four different types of solutions were prepared: (a) one with pure PEI (b) one with PEI and GNPs, (c) one with PEI and HAP, and (d) one with PEI and mixture of both nano-fillers (GNPs and HAP). The final concentration of GNPs and HAP into the final scaffold was selected to be 0.5 wt % and 1 wt % respectively. All PEI solutions, were characterized by low viscosity and such concentrations are expected to enhance the mechanical performance of the final scaffolds, and ultrafine fibers to be obtained. Blending of PEI polymer with mixture of both nano-fillers can improve the mechanical properties and the osteoinductive ability of the final scaffolds, making them more suitable for bone tissue engineering applications [46]. For comparison reasons, the total solid content of the nano-inclusions’ mixture in the final scaffold was kept constant at 0.5 wt % and 1 wt % respectively. The same nano-filler concentrations were also utilized in [43].

The selected solutions were prepared by stirring for 24 h at room temperature (RT) in order to achieve homogeneity. Then, each solution was loaded into a 3 mL syringe and was directly electrospun onto a grounded aluminum foil-covered collector (15 × 15 cm^2^). The deposition of fibers was carried out on a specific area of the collector, with dimensions 7.5 × 2.5 cm^2^, to obtain scaffolds with enough thickness in a short time period. The rest of collector’s surface was covered with Polytetrafluoroethylene (PTFE), in order to act as insulator and not to receive electrospun fibers. The solution flow rate for all samples was fixed at 6.6 mL/h and the applied voltage was kept constant at 13 kV in order to achieve continuous jet formation. The distance between the tip of the nozzle and the collector was set to 17 cm and a metallic, 18 gauge (G18) hypodermic needle (with inner diameter equal to 0.84 mm) was used. SEP was carried out at ambient conditions. For the needs of the present study, a lab-made SEP set-up has been utilized having a flat square aluminum plate as collector.

### 3.3. Scanning Electron Microscopy (SEM), Transmission Electron Microscopy (TEM), and Micro-Structure Analysis

The morphology and micro-structure of the fabricated scaffolds were evaluated by using SEM and TEM microscopy. For SEM, square strips having dimensions of 5×5 mm^2^ were machined and sputter coated with gold for 30 sec. Then, the samples were placed inside a Field Emission Scanning Electron Microscopy instrument (FE-SEM, FEI InspectTM F50) by using a Scanning Electron (SE) detector. The FE-SEM instrument operated at 5 kV. On the other hand, circular carbon coated copper grids (200 mesh) with diameter 3.05 mm were utilized for the TEM characterization experiments. These grids were positioned carefully on the collector’s surface, in order the electrospun fibers to be deposited during the SEP. The TEM instrument model was the JEM-2100/ HR-TEM, by Japan Electron Optics Laboratory (JEOL) and operated at 200 kV.

Fiber diameter measurements were obtained by image processing with ImageJ software (NIH, USA). Using different thresholds, SEM micrographs were converted to binary images. At least 150 fiber diameters were measured (50 measurements from three different samples’ images), while the average value and standard deviation (SSD) were reported. In addition, statistical test for the measured values of fiber diameter was carried out by using OriginPro with 95% confidence interval in order to determine fiber diameter distribution, which best describes experimental data. Three different goodness of fit tests were used: (a) Kolmoforov–Smirnov, (b) Kolmoforov–Smirnov (Modified), and (c) Anderson–Darling.

### 3.4. Porosity

The scaffold’s porosity was determined by using the ratio of the measured mass of the sample to the mass of a fully dense sample of the same size by measuring the sample’s dimensions (i.e., length, width, and thickness). The thickness of all scaffolds was measured with a thickness gauge by applying constant force. The porosity was determined by using Equation (1)

(1) $$P=\frac{M_{1}-M_{2}}{M_{1}}\cdot 100\;\left(\%\right)$$

where P is the porosity, M_1_ is the mass of a fully dense sample, and M_2_ is the mass of an electrospun scaffold. All utilized samples had the same dimensions for comparison reasons.

### 3.5. Static Water Contact Angle Assay

In order to evaluate surface hydrophilicity of the fabricated electrospun scaffolds, static water contact angle experiments were conducted by using a Contact Angle Goniometer apparatus (identification code: Kruss DSA100). Wetting characterization has significant effect in bone mechanics because it constitutes agent of biocompatibility. The utilized apparatus consists of a telescope equipped with protractor to measure the angle of the tangent at the three-phase contact point of the static drop by using a camera together with a Drop Shape Analysis Software. For the accurate wetting characterization of PEI scaffolds, a 29 gauge (G29) stainless-steel needle (having an inner diameter of 0.184 mm) controlled by a motor, in order to inspect the volume of the drop, and to avoid unwanted vibration. Taking into consideration scaffolds’ processing by hot-pressing, it was assumed that their surface had no roughness and then Young contact angle was measured. Measurements were taken at t = 0 s after a single droplet of bi distilled water (2 μL) got in contact with the surface of the fibrous scaffolds. Four measurements were taken for each sample and the experiments were performed at RT conditions.

### 3.6. Uniaxial Tensile Tests

Five rectangular samples of 30 mm length and 10 mm width were punched out from each scaffold type. The thickness of each specimen was measured to be approximately 0.2 ± 0.01 mm by using a thickness gauge. For all samples constant force was applied during measurements. Special care was taken during preparation to avoid severe damage of the samples. Duct tape was carefully placed at each edge of the samples to improve the mounting on the metallic grips of the testing device. The uniaxial micro-tensile tests were performed on Minimat 2000 (Rheometric Scientific, Shakopee, MN, USA) tensile instrument with 200 N load cell. During the experiments the span length was set at 12 mm. All experiments were performed at RT until sample’s failure at a strain rate of 5 mm/min. Prior testing, pre-tension was applied to all samples in order to ensure the extension and the receipt of load at the beginning of each experiment. The apparent mechanical properties of the scaffolds (apparent stress (σ) and strain (ε)) can be calculated at any time during the tensile experiments by using the following equations (Equations (2) and (3), respectively)

(2) $$\sigma =\frac{F}{A}$$

(3) $$\varepsilon =\frac{L-L_{o}}{L_{o}}$$

where F is the force, A is the cross-sectional area of the sample, L is the displacement, and L_o_ is the span length. The apparent mechanical properties of the scaffolds, ultimate tensile strength (σ_max_), young’s modulus (E), strain at break (ε_max_), yield stress (σ_y_), and yield strain (ε_y_) were measured and further analyzed.

## 4. Conclusions

In the present study, PEI scaffolds were successfully fabricated through SEP in order to future support three-dimensional tissue formation with perspective in orthopedic applications. More precisely, pristine PEI scaffolds and nano-modified ones with GNPs, HAP, and a mixture of both were fabricated for the needs of the present study. After fabrication, all scaffold types were further utilized to investigate its structural, physical, and mechanical properties. The structural properties of PEI scaffolds (i.e., calculation of mean fiber diameter and porosity) were determined by using SEM. In addition, contact angle experiments were conducted to investigate the surface hydrophilicity level of these scaffolds and their mechanical properties were investigated by conducting uniaxial tensile tests. After SEP, 3D fluffy structures were obtained and further processed by hot-pressing having an aim to enhance the mechanical properties of them. According to SEM, PEI fibers with no particular orientation were obtained while presented roughness on their surface. By nano-modification, scaffolds having thinner fibers were obtained with the HAP modified ones to exhibit the thinnest ones. Porosity value for all material sets was almost retained at the same levels apart from samples containing GNPs (12% reduction). Wetting characterization revealed the hydrophobic nature of the scaffolds (static contact angle values >90°). Nevertheless, nanoparticles presence enhanced the hydrophilicity of the scaffolds. Mechanical experiments in terms of uniaxial tensile tests that were conducted revealed that all modified PEI scaffolds exhibited enhanced mechanical properties with samples containing HAP to exhibit the best mechanical performance. In future work, the fabricated PEI scaffolds will be further investigated as potential host matrix material in order to support bone tissue formation.

## Abbreviations

SEP	Solution electrospinning process
ECM	Extracellular matrices
PEI	Polyetherimide
HAP	Hydroxyapatite
GNPs	Graphene nano-platelets
MWCNTs	Multi-walled carbon nanotubes
SEM	Scanning electron microscopy
TEM	Transmission electron microscopy
PCL	Polycaprolactone
THF	Tetrahydrofuran
DMF	Dimethylformamide
RT	Room temperature
PTFE	Polytetrafluoroethylene
FE-SEM	Field emission scanning electron microscopy
SE	Scanning electron
G	Gauge
JEOL	Japan electron optics laboratory
SSD	Standard deviation
P	Porosity
M_1_	Mass of a fully compacted sample
M_2_	Mass of an electrospun sample (scaffold)
σ	Stress
F	Force
A	Cross-sectional area
ε	Strain
L	Displacement
L_O_	Span length
E	Young’s modulus
σ_max_	Ultimate tensile strength
ε_max_	Elongation at break
σ_y_	Yield stress
ε_y_	Yield strain
SP	Supramolecular polymer
PMMA	Poly(methyl methacrylate)
